# Blowing Hot and Cold: Body Temperature and the Microbiome

**DOI:** 10.1128/mSystems.00707-21

**Published:** 2021-09-28

**Authors:** Kelsey E. Huus, Ruth E. Ley

**Affiliations:** a Department of Microbiome Science, Max Planck Institute for Developmental Biology, Tübingen, Germany; b Cluster of Excellence - Controlling Microbes to Fight Infections, University of Tübingen, Tübingen, Germany; University of California San Diego

**Keywords:** fever, human microbiome, microbiome, temperature, heat stress, hypothermia

## Abstract

The intestinal microbiome influences host health, and its responsiveness to diet and disease is increasingly well studied. However, our understanding of the factors driving microbiome variation remain limited. Temperature is a core factor that controls microbial growth, but its impact on the microbiome remains to be fully explored. Although commonly assumed to be a constant 37°C, normal body temperatures vary across the animal kingdom, while individual body temperature is affected by multiple factors, including circadian rhythm, age, environmental temperature stress, and immune activation. Changes in body temperature via hypo- and hyperthermia have been shown to influence the gut microbiota in a variety of animals, with consistent effects on community diversity and stability. It is known that temperature directly modulates the growth and virulence of gastrointestinal pathogens; however, the effect of temperature on gut commensals is not well studied. Further, body temperature can influence other host factors, such as appetite and immunity, with indirect effects on the microbiome. In this minireview, we discuss the evidence linking body temperature and the intestinal microbiome and their implications for microbiome function during hypothermia, heat stress, and fever.

## INTRODUCTION

Most animals, including humans, harbor complex intestinal microbiota that shape their metabolism and immune responses. Factors that affect microbial activity, such as nutrient, oxygen, and pH gradients, are key determinants of microbiome composition, function in the gastrointestinal tract, and can therefore indirectly influence host health; for example, the importance of diet and oxidative stress in microbiome-host interactions has been reviewed extensively elsewhere (1, 2). However, other factors known to influence microbial growth have been comparatively neglected in the microbiome field. Notably, despite the fundamental importance of temperature in controlling microbial growth and activity, relatively little attention has been paid to the influence of body temperature on the intestinal microbiome.

Although humans and mice maintain an average core body temperature of approximately 37°C, there is significant variation in body temperature across the animal kingdom (Table 1). Such variation may have implications for the transmission and evolution of intestinal microbes. Furthermore, fluctuations in an individual’s body temperature due to changes in environmental temperature, metabolic exertion, or immunological fevers have the potential to significantly alter the gastrointestinal niche. The body temperatures of ectothermic animals, such as reptiles, amphibians, fish, and insects, are especially susceptible to environmental fluctuations; intestinal microbiomes in these animals experience daily and seasonal fluctuations (3). Temperature thus has the potential to shape microbiome function in such diverse contexts as fever and climate change. In this minireview, we summarize the existing literature informing the relationship between body temperature and the gut microbiome and point toward interesting areas for future research.

**TABLE 1:** Diverse body temperature ranges in endotherms

Animal	Order	Normothermic temp(s) (°C)	Hypothermic temp(s) (°C)^*c*^	Hyperthermic temp(s) (°C)^*d*^	Reference(s)
Platypus	Monotremata	29–33	23 < 15 (HI)	35–38	82, 83
Armadillo	Cingulata	32–35	<25 (HI)	36–40	71, 84, 85
Human	Primata	35.7–37.3^*a*^; 36.2–37.5^*b*^	<35	>40 37.9–41 (FE)	73, 86, 87
Mouse	Rodentia	36.5–37.2	31–34	38–42; 37.8–39.3 (FE)	26, 56, 61
Rat	Rodentia	37.0–38.2	32–36	38.6–39.4 (FE)	56, 88
Bat	Chiroptera	35.5–37	≤5.8 (HI)	37.4–42	62, 69, 89
Pig	Artiodactyla	39.3–39.9	35–38	40.5–41.1	56, 90
Chicken	Galliformes	41.1–41.6	≤40	41.8–44.9 42.3–43 (FE)	91 – 94
Red-billed quelea	Passeriformes	40–41.8	NA	48–49.1	63

a Industrial.

b Historic.

c HI, hibernation; NA, not available.

d FE, fever.

## MICROBIAL RESPONSES TO TEMPERATURE

The building blocks of life are inherently sensitive to temperature. When it is too warm, proteins are denatured, nucleic acids lose their base pairing, and plasma membranes become excessively fluid. When it is too cold, everything slows down: enzymes work inefficiently, nucleic acids form inconvenient secondary structures, and plasma membranes are stiff. Microbes and other organisms have therefore adapted their cellular processes to grow within a specific temperature range and to respond to temperature stress beyond their optima (4).

The heat shock response is a conserved regulatory network found in all branches in life. Heat shock proteins include chaperones that stabilize and refold denatured proteins, and ATP-dependent proteases that degrade the misfolded proteins (5, 6). The complementary cold shock response is characterized by nucleic acid chaperones that prevent the formation of secondary structures in mRNA (7). Microbes also employ regulatory switches to control gene expression in response to temperature, thus adapting their activity to their environment (8, 9). In microbial communities subjected directly to fluctuating environmental temperature, such as coral reefs and compost heaps, temperature is well known to shape succession dynamics and metabolic activity (10, 11). In corals, environmental temperature further influences susceptibility to bacterial pathogens (12).

How resilient are intestinal microorganisms to temperature stress? *Escherichia coli* and other intestinal *Enterobacteriaceae* members are thermotolerant; many species in this family survive well at temperatures both cooler and warmer than those of the typical endothermic host. Enteropathogenic *Yersinia*, for example, will continue to grow at temperatures near 0°C (7), while lab strains of E. coli will grow from approximately 8°C (13) to 42°C and readily evolve to grow at temperatures up to 48°C or higher (14). Indeed, members of the *Proteobacteria* phylum are considered to be functionally flexible in response to many environmental stresses. Moreover, pathogenic members of this group, such as Salmonella, *Yersinia*, Pseudomonas, and pathogenic E. coli, explicitly respond to host temperature, using it as an environmental cue to upregulate virulence genes (8, 15, 16). These temperature-responsive genes tend to be even more strongly upregulated at fever-like temperatures of 42°C than at 37°C (15), and a temperature-responsive enzyme in P. aeruginosa likewise shows increasing efficiency up to 45°C (16). Clostridioides difficile, another major human intestinal pathogen, grows equally well at 37°C and 41°C *in vitro* (17, 18). Together, these observations indicate that intestinal pathogens both tolerate and exploit host temperature changes.

It is not clear, however, whether resident commensals exhibit the same resilience. A recent study demonstrated that bumblebee gut commensals varied in their preferred thermal niches (19), but growth rates beyond 37°C are uncharacterized for the majority of intestinal bacterial species. Many gut species are notoriously fastidious or not yet culturable and may not be nearly so permissive as pathogens in their growth temperature. Indeed, genomic studies of classic intestinal commensals, such as *Bifidobacterium* spp., point to a significant loss of heat shock response genes compared to those of their environmental relatives, likely reflecting adaptation to this relatively thermostable niche (20). At the most extreme end of the spectrum, many obligate intracellular symbionts in insect species have become extremely thermosensitive due to progressive genomic reductions during coevolution (21–23). Furthermore, nutrient availability, metabolic adaptations, and antibiotic resistance can modulate the thermal sensitivity of bacteria (6, 24, 25), and these factors vary meaningfully in a gut environment. Overall, therefore, temperature has significant potential to affect the growth and activities of intestinal microbes. Better characterization of the temperature sensitivities of intestinal commensals will be necessary to understand the shifts in microbiota communities upon thermal stress, as discussed in the following sections.

## ENVIRONMENTAL COLD STRESS AND THE INTESTINAL MICROBIOME

A recent body of literature has explored the responsiveness of the mammalian gut microbiota to cold exposure and host hypothermia. The abundance and diversity of *Lachnospiraceae* and the production of short-chain fatty acids (SCFA) increase consistently in response to decreased body temperature in rodents and humans (26–29). Hypothermic mice also have better metabolic health and are less susceptible to high-fat-diet-induced obesity, and these traits can be replicated by fecal-microbiota transplantation into mice kept at room temperature (28, 30, 31). However, overall alpha diversity decreases in cold-stressed rodents (26–28, 30) and fish (32), and certain gut species become undetectable, suggesting that some members of the microbiota are susceptible to cold stress. Interestingly, selective breeding of fish for cold tolerance led to microbiomes that were less diverse at baseline and less affected by cold temperature shock, suggesting that selection for a cold-adapted microbiome can occur over several generations (32).

Remarkably, there is substantial evidence that the microbiota not only responds to hypothermia but also affects host thermogenesis. During cold stress, mammals produce body heat primarily through nonshivering thermogenesis, a metabolic process occurring in brown adipose tissue (33). During nonshivering thermogenesis, the host mitochondrial protein UCP1 (uncoupling protein 1) uncouples the transport of protons from the synthesis of ATP, resulting in heat production (33). Mammals that lack a microbiota (due to germfree conditions or antibiotic treatment) have a cooler body temperature at baseline and experience worse hypothermia upon cold exposure (26, 34–36). This is likely because the microbiota, when present, improves dietary energy harvest for thermogenesis (26, 29, 34, 35). Indeed, a similar decrease in body temperature is seen in fasted animals (26). Increased SCFA production in cold-stressed conventionally raised animals appears to be a result of increased food intake, thus providing even more fuel for microbial metabolism and ultimately for host thermogenesis (29, 30). Indeed, oral gavage with SCFA, the energy-dense products of bacterial fermentation, rescues thermoregulation in antibiotic-treated animals (29, 36). When feces were transplanted from hypothermic to healthy rodents as described above, SCFA production and thermoregulatory capacity also improved (26). Although these studies focused largely on host thermogenesis via UCP1, it is notable that microbial fermentation of fiber also produces metabolic heat directly. This phenomenon is readily observed, for example, in compost heaps (11), and fermentation of ruminal contents produces measurable heat *ex vivo* (37). Fermentation-derived heat likely contributes to host temperature flux; it has been estimated that the human gut microbiota produces 60 kcal/h of heat during fermentation or approximately 70% of the total heat production of a resting individual (38). Together, therefore, these findings indicate that the metabolic activity of the microbiota helps to promote host thermogenesis.

## ENVIRONMENTAL HEAT STRESS AND THE INTESTINAL MICROBIOME

In contrast to the increased abundance of *Firmicutes* and SCFA observed during cold stress in mammals, multiple studies suggest that intestinal *Firmicutes* decline with heat stress (39–42), as does the overall alpha diversity of the gut microbiota. Strikingly, this decline has been observed across a wide variety of hosts, including both ectothermic and endothermic animals (3). Collectively, these data suggest a consistent impact of body temperature on intestinal *Firmicutes*, caused either by temperature itself or by conserved changes in host appetite or metabolism (3). In an elegant study that profiled the gut microbiota longitudinally over repeated cycles of heat stress in gerbils, both core body temperature and appetite cycled consistently with ambient temperature; so too did the abundances of several bacterial species and the production of SCFA (36). Moreover, a decline in microbiota alpha diversity (the taxonomic diversity or richness of a community) occurred in gerbils after repeated cycles of heat stress, indicating that the consequences of heat stress may accumulate over time (36).

Ectothermic animals are particularly sensitive to environmental heat stress; this represents a major conservation challenge in the face of climate change. Their microbiotas are also heat sensitive, which may impact host resilience to temperature stress. Intestinal alpha diversity and *Firmicutes* abundance decline sharply in lizards and amphibians exposed to heat stress, as does the temporal stability of the microbiota (39, 41, 43, 44). Loss of diversity persists months after the heat stress itself (43) and is associated with decreased digestive efficiency (44). Furthermore, the bacterial symbionts of several insects collapse entirely under heat stress, with severe consequences for host vitality (21–23, 45). Importantly, a heat shock gene variant in *Buchnera*, an obligate intracellular aphid symbiont, mediated the temperature sensitivity of the entire organism, including decreased fertility of the aphid during heat stress (23). This suggests that temperature can directly affect bacterial symbionts with consequences for the host, rather than being restricted to top-down host effects on the symbiont. Of course, nonintestinal microbial associations, such as those of corals, are also well documented to be sensitive to temperature increases as a direct result of heat stress (10, 46).

Endothermic animals are also affected by environmental heat stress. Livestock, for example, experience frequent heat stress under high-intensity farming practices, with negative consequences on animal health and on agricultural productivity. Cows, pigs, and chickens kept in hot and crowded conditions exhibit reduced food intake, reduced growth, increased intestinal permeability, and increased risk of systemic infection (47, 48). Changes in microbiota composition have also been reported in heat-stressed livestock, including the aforementioned declines in alpha diversity and *Firmicutes* abundance (40, 42, 49) and increases in relative abundances of *Proteobacteria* (50). These consequences are not limited to cows and chickens; heat stress also increases bacterial translocation and septic shock risk in humans (51), and hot summer weather is associated with an increased prevalence of Gram-negative bacterial infections (52–54). Concerningly, the prevalence and severity of heat stroke are increasing in both human and animal populations as a result of climate change (55).

## IMMUNOLOGICAL HEAT STRESS AND THE INTESTINAL MICROBIOME

In response to infection and innate immune stimulation, most mammals experience fever: a deliberately increased body temperature (Table 1). During fever, the hypothalamus responds to increased prostaglandin levels (an inflammatory lipid mediator released by stimulated macrophages) by triggering nonshivering thermogenesis in brown adipose tissue, thus generating metabolic heat via the same basic mechanism as during hypothermia. Even ectotherms will alter their behavior to seek out warmer temperatures during infection; indeed, a 1.5 to 5°C increase in body temperature in response to infection is remarkably prevalent across the animal kingdom (56). The presumed evolutionary benefit of this increase in core body temperature is to restrict the activity of pathogenic microbes, either via direct inhibition of microbial growth or via stimulation of immune responses at warmer temperatures (56). Both experimental and observational evidence suggests that fever responses do indeed help clear infections more quickly (56). Before the development of antibiotics, pyrotherapy was even used in humans to treat symptoms of syphilis (57).

Curiously, despite the impact of fever on infectious disease, we know little to nothing about what happens to the gut microbiota as a consequence of fever. In general, the innate inflammatory response is associated with increased levels of intestinal *Proteobacteria* and decreased alpha diversity, particularly when inflammation becomes chronic (2). However, the impact of acute fever responses on the mammalian microbiota is relatively unknown. A recent study found that patients infected with severe acute respiratory syndrome coronavirus 2 (SARS-Cov2) show fever-dependent shifts in the gut microbiota, including an increase in bacterial heat shock proteins, suggesting that fever temperatures do impact the human microbiome (58).

Although incapable of producing an internal “fever,” bumblebees use behavioral approaches, such as wing activity, to maintain high body and hive temperatures. These high temperatures have been shown to decrease pathogenic infections by directly modulating the activities of both the pathogen and the microbiota (59, 60). The bumblebee commensal *Lactobacillus* tolerates higher body temperatures than the parasite *Crithidia bombi*; moreover, *Lactobacillus* has an increased metabolic activity and thus higher antiparasitic activity against *C. bombi* at these high temperatures (60). Warmer bees thus have more intestinal *Lactobacillus* organisms and are more protected against infection (59).

Interestingly, the ability of the microbiota itself to increase host body temperature is observed during heat stress as well as during cold stress; antibiotic-treated mice maintain cooler body temperatures when hyperthermia is induced by the drug methylenedioxymethamphetamine (also known as ecstasy) (61). These data are consistent with a role for the microbiota in promoting metabolic heat production, rather than having a unique thermoregulatory capacity *per se*.

## TEMPERATURE OPTIMA ACROSS THE TREE OF LIFE

Mice and primates share a core body temperature of approximately 37°C and are the focus of most intestinal microbiome research. However, 37°C is not a constant across the animal kingdom; in fact, the overall range in normal core body temperatures is at least 10°C even in endotherms (approximately 30 to 40°C) (Table 1) (4). In flighted animals, body temperature is moreover more dynamic; during flight versus rest, core body temperature can increase by several degrees owing to the high metabolic demands of flying (62). A small African songbird called the red-billed Quelae has a normal body temperature of 41°C but can survive a toasty 49.1°C during flight-induced hyperthermia (63). At the opposite end of the spectrum, many animals are capable of entering torpor or hibernation, during which body temperature can drop to below 5°C (64). This is associated with marked intestinal and microbiota changes, although the effect of temperature is difficult to separate from dietary restriction during hibernation (64). Interestingly, there is also a moderate association in general between body temperature and diet across the phylogenetic tree, with higher cellulose consumption being generally associated with warmer core body temperatures (4). As noted above, microbial fermentation of fiber is itself heat producing and can also promote host thermogenesis, which may help to explain this relationship; however, this has not been rigorously tested.

Bats and birds show a notable convergence of their intestinal microbiota profiles, despite being phylogenetically distant and having a diverse range of diets (65). Both taxonomic groups tend to possess a low overall microbial burden, low alpha diversity, and a high variability between individuals, as well as having a high relative abundance of *Proteobacteria* and potential pathogens (65–67). This association may be driven by various host factors shared by flighted animals (65); however, body temperature is one such factor that has not yet been explored. Recently, our lab reported a positive correlation between abundance of the thermophilic archaeon *Methanothermobacter* and host body temperature across a range of mammalian and avian hosts (68). Although this correlation was inseparable from host phylogeny and might therefore be confounded by other host properties, it supports the hypothesis that host body temperatures can play a role in shaping gut microbiota composition.

Notably, many important zoonotic pathogens have reservoirs in bats and birds. It has been proposed that the high rates of zoonotic viral transmission from bats is driven by their natural body temperature being closer to the febrile state of humans (62, 69). Indeed, experimental work suggests that body temperature affects pathogen transmission; viruses that jump species show a much longer lag time when transferred to hosts with higher body temperatures and a faster course of infection when transferred to hosts with lower body temperatures (70). Mycobacterium leprae, the causative agent of leprosy, also shows a distinctive thermal preference; it naturally circulates in armadillos, who maintain a relatively cool body temperature of 32 to 35°C, and for this reason, it preferentially infects human hands and feet due to the lower temperature of these body extremities (71). As noted above, bats harbor not only high viral loads but also high intestinal populations of *Proteobacteria* compared to those in other rodents, as well as higher levels of predicted pathogenic bacteria (65–67). It is therefore interesting to speculate that the “fever hypothesis,” although traditionally framed around viral pathogen load, might extend to the abundance of intestinal bacteria.

## TEMPERATURE AND TIME

Not only does body temperature vary across host phylogeny, it also fluctuates with time, even in endothermic hosts. Human body temperature cycles with a person’s Circadian rhythm and with daily activities, such as eating, exercising, and sleeping (33). Female body temperature fluctuates according to monthly hormonal cycles (72), and body temperature changes with age (older individuals tend to have a cooler body temperature and have difficulty conserving heat).

Fascinatingly, core human body temperature has also decreased significantly since the 19th century, to the extent that 37°C is no longer strictly accurate as the benchmark for human body temperature (73). In high-income countries, the average healthy body temperature is currently closer to 36.5°C (73). Declines in human body temperature have also been demonstrated to occur rapidly in a hunter-gatherer population undergoing industrialization (74). This slight but significant decline over time may reflect substantially reduced burdens of infectious disease and thus reductions in chronic immune activation (73), as well as diverse other factors, including changes in ambient temperature, physical activity, and prophylactic antibiotic use (74).

While currently unexplored, it is tempting to speculate that these differences in body temperature might influence some of the observed changes in microbiota composition with age, across populations, and over generational time. The effect of industrialization on “modern” microbiota diversity and the associated rises in metabolic diseases, autoimmunity, and allergy are currently areas of intense research interest (75). While numerous factors certainly contribute to such large population-level differences, alterations in core body temperature represent one testable hypothesis.

## TEMPERATURE AND EXPERIMENTAL WORK

Temperature is also an important and often-neglected experimental consideration. Mice, the workhorses of health science research, require an environmental temperature of approximately 30°C for comfortable thermoneutrality. Humans, while maintaining the same body temperature, are thermoneutral at a much lower environmental temperature (∼24°C). Thus, although the majority of animal facilities operate at “room temperature,” research mice experience persistent cold stress under these conditions (76–78). This alteration in thermoregulation influences the basal metabolic rate and is therefore expected to affect all aspects of experimental results in mice, including microbiota findings. Notably, immune responses are significantly more robust in mice housed at warmer temperatures (76). Mice housed at room temperature become hypothermic rather than feverish in response to lipopolysaccharide (LPS) challenge; in contrast, mice housed at thermoneutrality exhibit robust fevers and clear infections more successfully (56, 76). This increased activity of immune responses at warmer temperatures is not unique to mice and has been observed in ectothermic animals (79) as well as in human immune cells *in vitro* (reviewed in reference 56), although there are clearly limits beyond which hotter temperatures are detrimental (56). Thus, our current knowledge of immune-microbial interactions, being drawn mainly from mice housed in conventional animal facilities, may be biased by a baseline state of cold stress and immunosuppression.

## IS BODY TEMPERATURE SEPARABLE FROM OTHER CONFOUNDERS? CONCLUSIONS AND CAVEATS

Both hot and cold stresses are associated with a loss of diversity and stability in the intestinal microbiotas of animals, as would be expected if these microbial communities are adapted to an optimal temperature. As noted recently (3), it is striking that intestinal *Firmicutes* abundances are negatively associated with body temperature, while intestinal *Proteobacteria* are positively associated with body temperature across a diverse range of hosts and contexts. These trends are observed in both ectotherms and endotherms experiencing temperature stress (3) but also in species with different core body temperatures. On the one hand, this might reflect the altered activity of host metabolism and immunity at different temperatures. Notably, since appetite is highly responsive to temperature stress (increasing in hypothermic animals and declining in hyperthermic or feverish animals) (80), it is difficult to separate the impact of body temperature from the changes in nutrient availability in the gut. Intestinal permeability and blood flow are also affected by temperature stress (48, 81); so too are immune responses (76). Intriguingly, however, flighted animals with a high body temperature show “heat stress” microbiota features while maintaining robust food intake and dampened inflammatory responses (65, 69). No study has yet examined the direct responsiveness of mammalian gut microbes to temperature stress or successfully uncoupled appetite, immunity, and temperature in animals. Further experimental work is therefore needed to clarify the mechanisms driving temperature-microbiota associations.

Although challenging, defining the relationship between body temperature and intestinal microbiota would have significant implications for our understanding of microbiota perturbations during fever and infectious disease, during climate-induced heat stress, across experimental studies in mice, and over generational changes in core human body temperature. Temperature undoubtedly affects the growth and metabolism of all living organisms; the role of body temperature in microbiota function thus represents a rich research area to be explored.
